# Editorial: Personalized health and precision medicine in practice

**DOI:** 10.3389/fsoc.2024.1367791

**Published:** 2024-02-19

**Authors:** Luca Chiapperino, Sylvain Besle, Séverine Louvel, Francesco Panese

**Affiliations:** ^1^STS Lab, Institute of Social Sciences, Faculty of Social and Political Sciences, Université de Lausanne, Lausanne, Switzerland; ^2^Centre de Recherche en Cancérologie de Lyon, INSERM U1052-CNRS UMR5286, Centre Léon Bérard, Université de Lyon, Université Claude Bernard Lyon1, Lyon, France; ^3^Université Grenoble Alpes, CNRS, Sciences Po Grenoble, Pacte, Grenoble, France

**Keywords:** personalized and precision medicine (PPM), precision medicine, personalized health, big data medicine, interdisciplinary collaboration, science and technology studies (STS), ethical, legal and social aspects (ELSA)

Social scientists have scrutinized extensively so-called personalized health and precision medicine. “Precision” and “personalization,” these scholars argued, are buzzwords of biomedical research: at best, they are umbrella terms that cover a diverse array of practices, technoscientific innovations, and biomedical scenarios (Abettan, 2016). At worst, these terms conceal perilous rhetoric: they operate a worrying brokerage of scientific promises and political reform of healthcare that make up a social meaning of innovation disjoined from its value for patients and the healthcare system (Prainsack, 2017). More recently, social studies of biomedicine have addressed the practical settings (e.g., public health; clinical sciences; environmental health sciences, etc.) in which normative, practical, organizational and technoscientific processes related to “precision” and “personalization” take place (Bourret and Cambrosio, 2019; Chiapperino et al., 2020; Bourret et al., 2021; Crabu, 2021; Polk et al., 2023). Leaving aside the question as to whether innovations will deliver on their promises, offers the opportunity for a different social and humanistic scrutiny of contemporary biomedicine: what are the specific institutional, local, practical, technical and scientific reconfigurations of health and medicine required by “precision” and “personalization?”

This Research Topic dissects how personalized health and precision medicine happen in these kinds of practices. Taken together, the nine articles composing the Research Topic demonstrate how these sociotechnical configurations are a diverse patchwork of healthcare experimentations and innovations in need of stabilization, validation, and standardization. Aspects concerning the implementation and validation of infrastructures of personalized health and precision medicine are best represented in the Research Topic. Taking personalized stem cell therapies for thalassemia as a case, Panwar documents the gamut of social, historical, ethnic and cultural categories that enter in the constitution of a biobanking infrastructure in Chennai, India. Caught up with considerations of caste, language and family relations, the personalization enacted in this biobanking infrastructure resembles a searching for definition of community and collective identity, rather than a technical process of biomedical research. Bühler analyzes the assemblage of a human biomonitoring cohort in Switzerland. Upstream from the detailed personalization of public health interventions for Swiss citizens lies the implementation of a longitudinal population-based cohort that can offer the tools, data and knowledge needed to assess the impact of the environment over health. The infrastructuration of precision public health, Bühler argues, rests upon challenging enactments of biosocial complexity in postgenomic sciences; that is, on the pragmatic and strategic choices made by scientists to render the complexity of social conditions, environments, relations and experiences amenable to biomedical research.

Another subset of articles in the Research Topic shows how the infrastructures and sociotechnical configurations of personalized health and medicine are no less dependent on often-neglected human labor. As argued by von Arx, data-intensive medical practices in cardiology may have profound implications not just for how, but also as to when a diagnosis is made. Her case study of remote cardiac monitoring illustrates the relevance of time in the development of precision cardiology: the alleged immediacy and continuity of telemedical devices does not automatically translate in a medicine of anticipation and early diagnosis. Rather, this promise of immediacy clashes with the need for human synchronization of these technologies: without adapting to the temporalities of the nurse ward the data-intensive monitor cannot produce meaningful knowledge of the patient's heart condition. Froger-Lefebvre et al. observe the same need for human labor and collective action. The authors analyze the implementation of an electronic prescription software for the use of genomic analyses in France. Vital to the introduction of this software is (what the authors qualify as) the dirty work of invisible professions: administrative and time-consuming tasks, such as the tuning of the new software into existing software, or the adaptation of established logistical workflows. All this essential work is often performed by overqualified workers in precarious jobs and raises profound questions about the invisible organizational choices and hierarchies of professions in precision medicine.

The political dimensions of practices developing personalized health and precision medicine are fleshed out more explicitly in two further contributions to the Research Topic. Pillayre and Besle analyze the mixed biomedical, organizational, and political aspects that partake to the definition of “rarity” in oncology. Documenting the constitution of validated lists of rare tumoral entities, the authors unpack a recursive tension in this process. On the one hand, the definition of rare cancers is intimately connected to the rise of genomic technologies: a growing number of cancers can in fact be defined as rare due to the availability of technologies that can measure their unique biological characteristics. On the other hand, rarity merely depends on negotiations between medical and political actors aiming to affirm the relevance of these clinical entities and direct toward them expert work in the international community. Pinel et al. emphasize instead how the development of epigenetic tests, known as biological clocks, renders specific forms of decay observable and socially relevant. Researchers assemble these tests by selecting specific sets of data and resources. In doing so, they also produce the relevance of specific collective approaches to aging in science and society. While the biological clock portrays aging as inevitable decline in the laboratory, the clock's transition into the market transforms aging into a modifiable trajectory, which demands action from allegedly empowered individuals and health consumers.

Of note is also the heterogeneity of the contributors to the Research Topic, including expertise in law, sociology, philosophy and anthropology, but also public health, epidemiology, clinical genetics and data sciences. This testifies to two intersecting points. On the one hand, this heterogeneity illustrates the topicality and relevance of STS analyses for the actors who are actively pursuing this new kind of medicine, healthcare and health promotion. As exemplified by the article of Walton and Christensen—two authors who can claim decades-long professional experiences in the development of genomic medicine—the change required to bring the tools of genomics in healthcare systems are neither merely technological nor just clinical or scientific. The authors underline the often-neglected social, organizational and policy dimensions of the genomic transformation of medicine. Hospital workflows, institutional standards, billing procedures, professional education or even the architectures of hospitals should be the target of change in the healthcare system to accelerate the use of genomics in medicine. And the importance of bringing “the social” into the development of personalized health and precision medicine is of no less relevance at an epistemic level to these actors. As pointed out by the perspective article of Delpierre and Lefèvre—two public health scholars—models of personalization are too tied to a biomedical model of health, which often neglects the interactions between the environmental, socio-economic, psychological, and biological determinants of health. Drawing upon a biopsychosocial model of medicine, the authors underline that biomedical knowledge of health—based for instance on the tools of “omics sciences” (e.g., genomics, transcriptomics, epigenomics, proteomics, metabolomics, and pharmacogenomics)—may not be fit for the purpose of delivering on the promises of personalization. Personalized medicine, they conclude provocatively, should be tantamount to a serious consideration of the “person” beyond the unique biological characteristics of individual patients.

On the other hand, the heterogeneity of contributions to our Research Topic points to the reflexivity on the epistemic, technical, organizational, regulatory and political dimensions of personalized health and precision medicine that can come from within biomedical practices (see Mann and Chiapperino, 2023). If anything, the Research Topic is thus a reminder of the importance of engaging with a diverse array of perspectives on personalized health and precision medicine and actors that should include citizens and patients who are the primary targets of these innovations. As Berti Suman et al. argue in their perspective article, grassroots-driven initiatives—which the authors call “personalization from below”—could help identify and develop alternative understandings of “personalization” and “precision” for the future of medicine. Reaching beyond the model of public engagement with biomedicine, their political argument for personalization from below rests upon epistemic, democratic and equality considerations that heavily challenge the hierarchical structures often dominating healthcare.

In summary, the results of our Research Topic point to the openness and situatedness of the sociotechnical configurations labeled as precision medicine and/or personalized health, and to the much-needed involvement of heterogeneous actors (e.g., civic associations, activists, citizens, patients, researchers, healthcare professionals, and policymakers) in their making. Moreover, we believe that the Research Topic also offers a clear rationale to integrate social studies of personalized health and precision medicine with the debates on these matters internal to biomedical sciences. Future studies taking a cross-cutting, interdisciplinary, and collaborative approach may be key to realize the model of “personalization” and “precision” *we*—as a heterogeneous set of scholars and citizens—want (Prainsack, 2014): a model that is both challenging to existing hierarchies in healthcare and is attuned to its practical conditions of possibility.

## Author contributions

LC: Conceptualization, Supervision, Validation, Writing—original draft, Writing—review & editing, Funding acquisition. SB: Funding acquisition, Supervision, Validation, Writing—review & editing. SL: Funding acquisition, Supervision, Validation, Writing—review & editing. FP: Conceptualization, Funding acquisition, Supervision, Validation, Writing—review & editing.
